# Managing growing teratoma syndrome: new insights and clinical applications

**DOI:** 10.2144/fsoa-2019-0075

**Published:** 2019-10-10

**Authors:** Srdjan Saso, Nicolas Galazis, Christos Iacovou, Kleio Kappatou, Menelaos Tzafetas, Benjamin Jones, Joseph Yazbek, Konstantinos Lathouras, Jonathan Anderson, Long R Jiao, Richard J Smith

**Affiliations:** 1 Department of Gynaecologic Oncology, Queen Charlotte's & Chelsea Hospital, Imperial College Healthcare NHS Trust, Du Cane Road, London W12 0NN, UK; 2 Department of Surgery & Cancer, Institute of Reproductive & Developmental Biology, Imperial College London, Hammersmith Hospital Campus, Du Cane Road, London, W12 0NN, UK; 3 Department of Cardiothoracic Surgery, Division of Surgery & Cancer, Imperial College London, Hammersmith Hospital Campus, Du Cane Road, London, W12 0NN, UK; 4 Department of Hepatobiliary Surgery, Division of Surgery & Cancer, Imperial College London, Hammersmith Hospital Campus, Du Cane Road, London, W12 0NN, UK

**Keywords:** chemotherapy, immature teratoma, mature teratoma, oncology

## Abstract

Although a recognized condition, growing teratoma syndrome (GTS) has no guidelines for management, and patients diagnosed with the condition are managed empirically by the most appropriate teams. We report a case of GTS in a 33-year-old patient who was initially treated with unilateral salpingo-oophorectomy and subsequent chemotherapy for a germ cell ovarian tumor. GTS was subsequently diagnosed with massive pelvic and upper abdominal masses as well as lung tumors. We also conducted a literature review on cases of GTS presenting with large tumors. Based on this, we suggest a management plan to guide the care of women with GTS. The condition is best managed in a multidisciplinary team involving the relevant surgeons, including gynecologist, abdominal and thoracic surgeons.

## Lay abstract

Growing teratoma syndrome (GTS) is a rare but recognized condition presenting with an abdominal mass in a woman with history of malignant ovarian tumor treated with chemotherapy. There are no established guidelines for the management of women diagnosed with GTS. We report a case of GTS in a 33-year-old patient requiring major abdominal and thoracic surgery. We also review the literature on previously published cases of GTS and propose a management plan to guide clinicians looking after women with GTS. Collaboration between different surgeons including gynecologists, abdominal and thoracic surgeons as well as radiologists and oncologist should be sought.

Growing teratoma syndrome (GTS) is a rare condition, which presents with benign (mature) teratoma masses either during or after chemotherapy [1]. It is associated with a nonseminomatous germ cell tumor (NSGCT) of the testis or the ovary [1]. The incidence of GTS is reported at 1.9–7.6% after testicular and 12% after ovarian NSGCT [2]. GTS has been increasingly discussed in the literature owing to better awareness of this clinical entity and the improvement in diagnostic imaging tools [3]. Early diagnosis of GTS is vital as it reduces the risk of extensive surgery, as well as morbidity to the patient [3].

Diagnostic criteria of GTS include: normalization of serum tumor markers such as α-fetoprotein (AFP) and human chorionic gonadotropin; visualization of enlarging or new masses despite appropriate chemotherapy for NSGCT; and exclusive presence of mature teratoma only in the resected specimen [1]. The exact pathophysiology of GTS remains unknown. To date, two theories have been described: destruction of the malignant (immature) components of the teratoma and sparing of the benign (mature) components during chemotherapy; chemotherapy-induced transformation of immature to mature cells [3–5].

In order to highlight current insights into GTS management, we report a case of GTS, presenting with a large pelvic mass 2 years after chemotherapy for immature teratoma. We defined ‘large’ as a mass measuring above 10 cm in at least one dimension. We also conducted a literature review on cases of GTS presenting with large tumors. We chose this subgroup as tumors of this size are more challenging to manage surgically.

Our aim was to improve our understanding of this rare condition and identify strategies to assist in earlier diagnosis and management.

## Case report

A 33-year-old multiparous woman of Indian descent presented in July 2009 to a district general hospital with abdominal bloating and pain. The patient underwent a transvaginal ultrasound scan, which showed a 12-cm left adnexal lesion with suspicious features, namely papillary projections, multiple loculations and increased vascularity. AFP was raised at 1100 ng/ml. The above prompted referral to a tertiary center and an urgent computed tomography (CT) of the chest, abdomen and pelvis did not show evidence of peritoneal or distant metastasis. Fertility-sparing surgery was performed with an open left salpingo-oophorectomy, omentectomy and peritoneal washings in September 2009. There was no macroscopic disease in the abdomen but the ovarian capsule was breached. Macroscopic clearance was achieved during surgery. Histology confirmed mixed germ cell tumor, consisting of a high-grade immature teratoma and a yolk sac tumor. Radiological and surgical staging were consistent with Stage 1C.

Because of persistently elevated AFP levels postoperatively, she underwent two cycles of chemotherapy consisting of bleomycin, etoposide and platinum. Disease progression was noted during these two cycles so her chemotherapy was switched to irinotecan, paclitaxel and oxaliplatin for further four cycles. CT at the end of chemotherapy revealed multiple cystic/solid lesions in the pelvis (arising from the right ovary), abdomen (involving the liver and gall bladder) and lungs, which at that stage, were deemed inoperable. Positron emission tomography did not reveal any hot spots, and tumor markers were normal. At that stage, the patient did not complain any relevant symptoms and for this reason, she was managed expectantly.

A year later, she presented with abdominal distension. A 20 cm pelvic mass arising from the right ovary was shown on CT. Given the size and location of the lesions, the decision was made to proceed with hemi-hepatectomy, cholecystectomy, resection of right hemidiaphragm and left lung lobectomy. The multidisciplinary team deemed surgical intervention was required to obtain a histological diagnosis and prevent morbidity due to pressure of vital structures. No gynecological surgery was performed at that stage because of the extent of the upper abdominal and thoracic surgery. All specimens showed a benign mature cystic teratoma. Three months later, completion debulking surgery was performed involving the removal of the uterus, remaining right tube and ovary, omentum and spleen. Again, histology showed a mature cystic teratoma in all specimens.

Three years later (in July 2013), the patient underwent another operation to remove an anterior abdominal wall lesion, which was also reported as mature cystic teratoma on histology. Ten years later, in 2019, the patient is on hormone-replacement therapy, managing well with no evidence of recurrence in subsequent scans and tumor markers.

## Discussion

Eleven case reports, published between 1983 and 2014, were identified in the literature where GTS presented with a tumor larger than 10 cm following chemotherapy for NSGCT (Table 1) [2,6–15]. With the exception of two reports, women were of child-bearing age [2,12]. Six studies reported the size of the primary immature teratoma; the average size was at least 20 cm [2,7–10,12]. This may suggest that a large primary tumor may predispose to subsequent GTS with a tumor over 10 cm. This is a premature assumption at this stage and will need to be validated by larger case series/cohort studies in the future. Our case is very rare as it involved extra-pelvic organs such as the liver, spleen and diaphragm. Moreover, to our knowledge, it is the first published case where GTS affected the lung.

**Table 1.** Growing teratoma syndrome: review of literature with cases >10 cm in size.

Number of cases	Study (year)	Age	Initial histo	Size of immature teratoma (cm)	Stage	Grade	Surgery	Chemotherapy	Tumor markers after primary treatment	Re-presentation (months)	Site	Size (cm)	Final histo	Surgery	Outcome
1	Aronowitz (1983)	15	Immature teratoma left ovary	N/R	IA	3	LSO and wedge biopsy of right ovary and omentum	Vin, Act, Cyclo (11)	N/R	12	Multicystic pelvic mass adjacent to right ovary	12	Mature teratoma	RSO, total hysterectomy and removal of pelvic mass	Alive
2	Itani (2002)	24	Immature teratoma right ovary	16 × 9	IC	N/R	Right adnexectomy and enucleation of cystic tumor of left ovary	BEP (3)	Negative	15	Right para-aortic lesion occupying all right retroperitoneal cavity	11 × 6	Mature teratoma	Cytoreductive surgery	Alive
3	Tangjitgamol (2006)	5	Immature teratoma ovary N/R	11	IA	3	SO	BEP (2) VAC (2)	Negative	5	16 cm tumor mass beneath the right diaphragm and tumor nodules in cul-de-sac	16	Mature teratoma	Surgical resection and debulking	Alive
4	Dewdney (2006)	19	Immature teratoma right ovary	30	N/R	3	Exploratory laparotomy	BEP (3)	N/R	8	Mid-abdominal mass encasing retroperitoneal vessels with internal calcifications and severe left hydronephrosis	25	Mature teratoma	Laparotomy and resection of mass and left-sided percutaneous nephrostomy	Alive
5	Malik (2008)	22	Immature teratoma left ovary	20 × 25	IA	3	LSO	Vin, Act, Cyclo (3)	Negative	36	Pelvis + abdomen	20 × 20	Mature teratoma	Mass resection after adhesiolysis and infracolic omentectomy	Alive
6	Rashmi (2010)	19	Immature teratoma left ovary	25 × 20	IA	3	LSO and omental Bx	3 cycles of chemo, type N/R	N/R	36	Pelvis + abdomen	20 × 20	Mature teratoma	Excision of tumor, adhesiolysis, omentectomy and peritoneal Bx	Alive
7	Sengar (2010)	26	Mature and immature teratoma right ovary	N/R	IA	1	RSO, pelvic LND + omental Bx	BEP (3)	N/R	6	Pelvis + abdomen	17	Mature cystic teratoma	Excision of tumor from sigmoid colon, right pelvic nodule, AAW nodule and omentectomy	Alive
8	Mrabti (2011)	18	Immature teratoma right ovary	22 × 18	IC	2	TAH, RSO and omentectomy	*Cis* + Eto (6)	Negative	6	Pelvis + abdomen	25 × 21	Mature teratoma	Excision of tumor	Alive
9	Altinbas (2012)	52	Immature teratoma ovary N/R	N/R	3C	3	TAH, BSO, PA LND and omentectomy	BEP (6) Paclitaxel (3)	Negative	36	Liver, diaphragm, abdomen, pelvis, pelvic lymph nodes	20 × 10	Mature teratoma	Excision from liver, diaphragm, peritoneum, sigmoid colon, omentum and RP	Alive
10	Ohashi (2014)	30	Immature teratoma ovary N/R	N/R	3C	N/R	TAH + BSO	N/R	N/R	216	Pelvis + abdomen	40 × 40	Mature teratoma	Excision of tumor	Alive
11	Mir (2014)	16	Immature teratoma ovary N/R	N/R	N/R	N/R	TAH + BSO	BEP (3)	N/R	36	Abdomen	40 × 25	Mature teratoma	Excision of tumor + peritonectomy	Alive

AAW: Anterior abdominal wall; Act: Actinomycin D; BEP: Bleomycin, etoposide, cisplatin; BSO: Bilateral salpingo-oophorectomy; Bx: Biopsy; Cyclo: Cyclophosphamide; Eto: Etoposide; Histo: Histology; LND: Lymph node dissection; LSO: Left salpingo-oophorectomy; N/R: Not recorded; Omentect: Omentectomy; PA: Para-aortic; RP: Retroperitoneum; RSO: Right salpingo-oophorectomy; SO: Salpingo-oophorectomy; TAH: Total abdominal hysterectomy; VAC: Vinblas­tine, Adria­mycin, Cisplatin; Vin: Vincristine.

In all studies, the primary pathology was managed by debulking surgery. This was either unilateral salpingo-oophorectomy or hysterectomy with bilateral salpingo-oophorectomy followed by adjuvant chemotherapy [2,6–15]. The latter commonly involved three cycles, mostly bleomycin, etoposide and platinum. Tumor markers during or after chemotherapy were only reported in five studies and they were normal [2,7,9,12,13]. The average size of GTS was 20 cm (range: 11–40 cm) and all patients underwent debulking surgery confirming mature cystic teratoma. Prognosis was excellent with all patients found to be disease free at follow-up. However, no pregnancies have been reported after surgery for GTS [2,6–15]. In addition, significant patient morbidity has been described both from immediate and short-term surgical complications and the long terms effects of surgical menopause in young patients [2,6–15].

In our review, GTS seems to present at an average 15 months from chemotherapy. Li *et al*. who performed a review of all cases of GTS until February 2016 reported a mean interval of 27 months from chemotherapy [16]. This could potentially indicate that large initial NSGCTs (over 10 cm) may present with GTS earlier compared with smaller NSGCTs. Growth rates vary widely among patients with a median increase in diameter of 0.7 cm per month and median growth of volume of 12.9 ml/month [17]. Any patient of NSGCT with a rapid increase in tumor growth rate after chemotherapy in presence of normal serum markers should raise suspicion of GTS [18].

High index of suspicion and earlier diagnosis can have a significant effect on the management of the patient with GTS. Although a benign condition, GTS requires debulking surgery because of mechanical obstruction and pressure to vital structures [18]. The earlier the diagnosis, the less likely will be the need for radical surgery, which may involve permanent damage to the patient's reproductive potential. Furthermore, we suggest that in cases where debulking surgery is likely to cause infertility, the patient is referred urgently to a fertility specialist and a consideration of oocyte cryopreservation is made.

### Standardization of care

**Figure 1.**
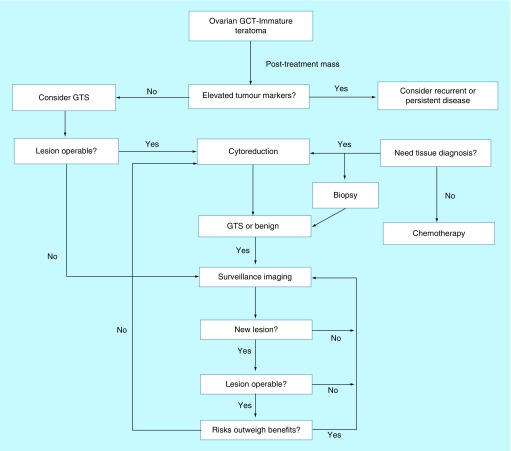
Algorithm of management of growing teratoma syndrome. GCT: Granulosa cell tumor; GTS: Growing teratoma syndrome.

Although a recognized entity, no consensus or guidelines on the management of GTS exists. The patients are managed empirically, usually by a multidisciplinary team of surgeons, oncologists and radiologists. Our literature review confirmed the young age, which NSGCT, and subsequently GTS, present (range 5–52 years, mean 22 years). This stresses the importance of early diagnosis and appropriate management to minimize the need for radical surgery and subsequent loss of fertility. We, therefore, propose an algorithm to guide the management of the condition (Figure 1).

### Diagnosis

We suggest three-monthly follow-up appointments with tumor markers and ultrasound examinations for the first year after treatment of NSGCT to ensure early detection of disease recurrence or development of GTS. Six-month surveillance may ensue for the second year, followed by yearly surveillance for at least further 3 years post-treatment. CT scan surveillance can be six monthly for the first year, and yearly from then on. CT imaging is a useful modality and demonstrates characteristics of radiographic maturation including increased density, better circumscribed margins, onset of internal calcifications, amalgamation of fat and solid/cystic components [19–21]. Positron emission tomography and ultrasonography have limited use in the detection of GTS [22,23].

### Management

Management plans following a positive CT scan should be made within a multidisciplinary team setting involving surgeons, oncologists and radiologists. Surgery is the mainstay of treatment, and histology confirms the diagnosis. Furthermore, it avoids the need for further chemotherapy, which may have an adverse effect to the patient's reproductive potential. Surgical exploration and complete resection when possible is suggested to reduce the risk of recurrence necessitating more radical surgery, which is associated with increased morbidity and mortality. Fertility-preserving surgery may be reasonable in selected women who have been adequately counseled [20]. Careful surveillance is recommended in these cases. When fertility is no longer desired, completion surgery can be performed in the presence of residual disease. Moreover, we suggest that the patient should be offered an appointment with a fertility specialist for consideration of assisted reproduction techniques to expedite pregnancy. Further options of fertility preservation also include embryo cryotherapy, oocyte cryopreservation, and ovarian autotransplantation [24].

### Follow-up & prognosis

With partial resection, the recurrence rate for GTS has been quoted between 72 and 83% while with complete resection, the recurrence is considerably reduced to 0–12.7% [17,19]. Malignant transformation of GTS has been documented in case reports at a rate of 3% [25,26]. GTS has an overall good prognosis with only a few reported deaths, which are mainly attributed to postoperative complications [5,17,22,27].

The 5-year overall survival rate of patients who had undergone surgery following GTS is 89% [5]. Recurrences of GTS have been reported up to 10 years from surgical management. Therefore, regular follow-up with CT scan is recommended; six-monthly for the first year and yearly thereafter for at least 4 more years [28,29].

## Conclusion

GTS is a rare benign condition, which occurs following chemotherapy for NSGCTs. Improved awareness and follow-up may lead to earlier diagnosis and subsequently less extensive surgery. The condition is best managed in a multidisciplinary team involving the relevant surgeons, including gynecologist, abdominal and thoracic surgeons. In addition, oncologists, radiologists and menopause and fertility specialists need to be involved in the management of these women who need to be counseled about the short- and long-term effects of debulking surgery for GTS.

## Future perspective

We envisage that the management of GTS will be standardized based on international guidelines and protocols, and centralized in tertiary centers. We expect that, as the scientific community is becoming more aware of this rare condition, large cohort studies will be conducted to further add to the pool of knowledge. The latter, as well as the management algorithm proposed in this review, should promote standardization of care of GTS.

**Executive summary**

- Growing teratoma syndrome (GTS) is a rare condition presenting with benign (mature) teratoma masses either during or after chemotherapy for malignant (immature) teratoma.
- Although a recognized condition, GTS has no management guidelines.
- In this paper, we report a case of GTS managed successfully with multiple surgeries under a multidisciplinary team of gynecological oncologists, hepatobiliary and thoracic surgeons.
- After a thorough literature review we are proposing an algorithm to guide clinicians in the management of GTS.
